# Synthesis and thermoelectric properties of InSb alloys by solid reaction

**DOI:** 10.1016/j.dib.2018.03.108

**Published:** 2018-03-30

**Authors:** Kang Wang, Peng Qin, Zhen-Hua Ge, Jing Feng

**Affiliations:** Faculty of Materials Science and Engineering, Kunming University of Science and Technology, China

## Abstract

The data presented in this article are related to the research article entitled” Synthesis and thermoelectric properties of InSb alloys by solid reaction” (Kang Wang, Peng Qin, Zhen-Hua Ge, Jing Feng ,2017) [1]. This article describes a new strategy of eutectic melting for improving the TE properties. We put the powders of In and Sb (both is 200 mesh) with a certain proportion of InSbx (*x* = 1.0, 1.01, 0.99, 0.97 and 0.95) and sealed them in different crucibles(23 mm × 18 mm).

**Specifications Table**

**Table:** 

Subject area	*Chemistry*
More specific subject area	*Thermoelectric materials*
Type of data	*Table, image (x-ray, microscopy), figure*
How data was acquired	*X-ray diffraction, EPMA,SEM*
Data format	*filtered, analyzed*
Experimental factors	*Put In powder and Sb powder in a certain proportion of InSbx (x = 1.0, 1.01, 0.99, 0.97 and 0.95) and sealed in different crucibles(23 mm × 18 mm).*
Experimental features	*The samples were melted at 973 K for 3 h under purified argon atmosphere in a tube furnace, then the furnace was cooling down to room temperature at the rate of 20°C/min.*
Data source location	*Kunming, China for collected samples/data if applicable*
Data accessibility	*The data are available with this article*

**Value of the data**

• The data presents the specific experimental process.
• The details of the experiment, test methods and instrument models.
• The data shows that the temperature dependence of the lattice thermal conductivity.

## 1 Data

**Fig. 1:**
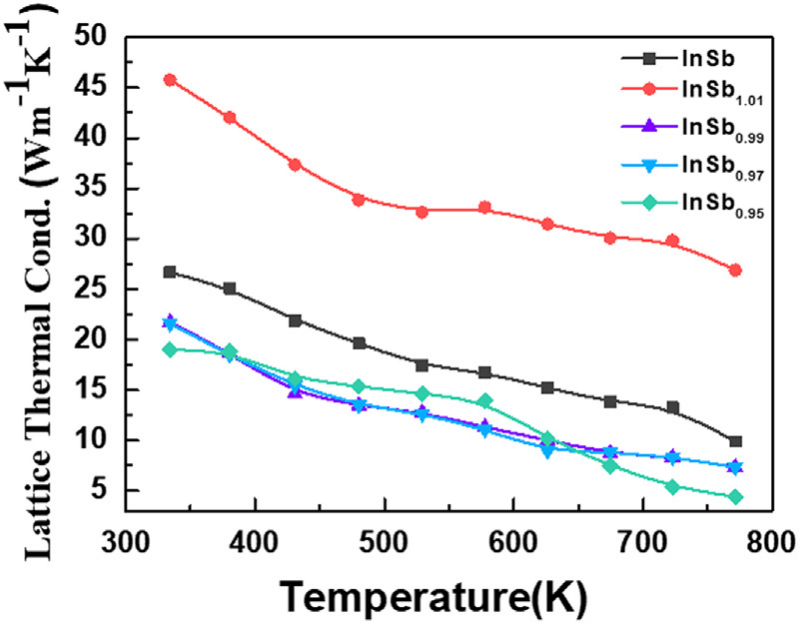
Temperature dependence of the lattice thermal conductivity of the InSb sample with different *x* contents.

The dataset of this article provides the sample preparation process information. The Fig. 1 show the temperature dependence of the lattice thermal conductivity of the InSb sample with different *x* contents. Table 1 shows the density of the InSb sample with different *x* contents.

**Table 1:** The density of the InSb sample with different *x* contents.

	**InSb**_**1.01**_	**InSb**	**InSb**_**0.99**_	**InSb**_**0.97**_	**InSb**_**0.95**_
***ρ*****(g/cm**^**3**^**)**	5.75	5.717	5.706	5.697	5.689

## 2 Experimental design, materials and methods

The InSb*_x_* samples with different *x* values (*x* = 1.01, 1.0, 0.99, 0.97 and 0.95) were prepared by using the In, Sb, (99.9% in purity) powders. We put the powders of In and Sb (both is 200 mesh) with a certain proportion of InSb*_x_* (*x* = 1.0, 1.01, 0.99, 0.97 and 0.95) and sealed them in different crucibles(23 mm × 18 mm). Then delicately placed the crucibles into quartz tubes. The samples were healed from room temperature to 973 K with the pressure at 5 × 10^−2^ Pa under purified argon atmosphere in 30 min. Then samples were melted at 973 K for 3 h and the furnace was cooling down to room temperature at the rate of 20°C/min. Finally, we obtained the cylindrical specimens with the size of 16 mm × 5 mm.

The speciments were characterized by X-ray diffraction (XRD: Cu-Kα Bruker D8, Germany) and the density (*ρ*) of the samples were determined by Archimedes method. The fractographs were measured by a field emission scanning electron microscope (SEM, ZEISS, EVO18-21-57), and the composition identification was conducted by electron probe microanalysis (EPMA; Rigaku, Tokyo, Japan) on a JEOL JXA-8230 microscope. To measure electrical conductivity and Seebeck coefficient, the samples were cut from the sintered pellets into rectangular bars with a dimension of 1 × 3 × 10 mm. The Seebeck coefficient and electrical resistance were measured using a Seebeck Coefficient/Electrical Resistance Measuring System (ZEM-3, UlvacRiko, Japan) in a helium atmosphere. The thermal diffusivity (*D*) was measured by laser flash method (NETZSCH, LFA457, Germany). The thermal conductivity (*κ*) was calculated by the density (*ρ*), specific heat and thermal diffusivity using the equation *κ* = *DC_p_ρ*. These thermoelectric properties were measured in the temperature range of 323–773 K.

The temperature dependences of $\kappa_{l}$ and $\kappa_{c}$ for the InSb samples and of $\kappa_{l}$ for InSb are shown in Fig. 3d and Fig. 1. The lattice thermal conductivity $\kappa_{l}$ was estimated by subtracting the carrier thermal conductivity $\kappa_{c}$ from κ using the Wiedemann–Franz relationship $\kappa_{c}=L\sigma T$, where *L* is the Lorentz number [2], [3].

This work mainly adopted the strategy of eutectic melting to prepare InSb type alloy thermoelectric material. Using solid reaction to prepare eutectic compounds not only can prepare lamellar InSb alloy, but also this method is efficient and simple. Furthermore, the thermoelectric properties of the alloy could be improved by doping and changing the experimental conditions.
